# Ring-Shaped Baffle Effect on Separation Performance of Lithium Carbonate Micro Particles in a Centrifugal Classifier

**DOI:** 10.3390/mi11110980

**Published:** 2020-10-30

**Authors:** Moonjeong Kim, Jemyung Cha, Jeung Sang Go

**Affiliations:** 1 School of Mechanical Engineering, Pusan National University, Busan 46241, Korea; mjkim80@pusan.ac.kr; 2 SEMES Co. Ltd., 77, 4sandan 5-gil, Jiksan-eup, Seobuk-gu, Cheonan-si, Chungcheongnam-do 31040, Korea

**Keywords:** centrifugal classifier, particle separation, lithium carbonate, Rosin-Rammler

## Abstract

In this work, a centrifugal classifier for separating lithium carbonate particles, used as a cathode material for lithium-ion batteries, was investigated. This work numerically evaluates the internal flow and particle separation performance of the centrifugal classifier. The complex turbulent flow field in the classifier is key to understanding particle motion. A Reynolds stress model, to describe air flow field, and a discrete phase model, to track particle motion, were applied to a numerical simulation. Design parameters such as mass flow rate and rotor speed were investigated, and a ring-shaped baffle, in particular, was designed to investigate the effects of flow and particle separation in the centrifugal classifier. The simple geometry of the baffle changes the movement direction of unseparated particles to the rotor cage region, and increases the local air velocity in the separation zone. The numerical analysis results were verified through a baffle experiment.

## 1. Introduction

In recent years, the use of electric vehicles and energy storage systems using lithium-ion batteries has been rapidly growing. Lithium carbonate (Li_2_CO_3_) is used as the cathode material in lithium-ion batteries; its particle size distribution (PSD) is directly related to the electrical performance of these batteries [1,2]. In general, this cathode material is prepared by processes such as weighing, mixing, calcining, crushing, classification, removing moisture, and packaging from raw materials. Among them, the classification process obtains a specific size range of fine particles from raw Li_2_CO_3_ particles.

In the classification process to separate mixed particles by size, it is important to analyze the complex physical interaction between the gas and particles. This study focuses on separation of raw Li_2_CO_3_ particles using a centrifugal classifier [3]. Numerical simulations of gas–particle two-phase flow were performed to investigate a complex flow field and the particle motion in the centrifugal classifier. The effects of design parameters, such as mass flow rate and rotor speed, on the particle separation performance were investigated. Additionally, the ring-shaped baffle was numerically designed to generate a strong velocity field in the direction of the rotor cage to increase separation throughput. Finally, experiments were conducted to validate the numerical results, using an industrial centrifugal classifier.

Numerical simulation using computational fluid dynamics (CFD), due to its extensive physical modelling capabilities, has been widely used to evaluate the classification performance of centrifugal classifiers. To improve the design of the centrifugal classifier, numerical simulation must accurately predict the particle separation performance, which depends on the modeling of fluid flow and particle behavior. In previous numerical studies, turbulence models such as RSM (Reynolds stress model) and LES (Large eddy simulation) were adopted to describe fluid flow, and the Lagrangian method, including a discrete phase model (DPM), was applied to predict the particle behavior. Through the numerical approach, the high swirling flow and characteristics of particle motion in the centrifugal classifier can be accurately captured.

In particular, many works on centrifugal classifiers have been devoted to improving the classification performance by optimizing the design parameters. Eswaraiah et al. performed a numerical simulation of the particle separation performance by optimizing the velocity distribution between blades installed in the rotor cage in the air classifier. The design variables influenced the strength of the centrifugal force acting on the particles, and the shape of the classification curve was varied [4,5,6,7]. Liu et al. focused on a uniform flow between the rotor cage and the guide vane to improve the classification performance. Axial inclined guide vanes were designed to avoid a reduction in separation efficiency [8,9,10]. Ren et al. conducted CFD simulation to study the flow field distribution in a rotor cage with non-radial arc blades. Using the modified rotor blades, the numerical results agreed well with the experimental results for classification accuracy [11]. Recently, Sun et al. investigated the effect of air-inlet direction on the classification performance, by using CFD simulation and a visualized vertical vortex in the conical part, and a horizontal vortex in the classifier chamber [12]. It is also worth noting that the selection of a suitable turbulence model is very important in predicting the turbulent flow of a centrifugal classifier. In the research of Vakamalla and Mangadoddy [13], a proposal was made to select suitable turbulence models by considering the turbulence levels, such as RSM for an industrial scale hydrocyclone with low turbulence levels, and LES for a small hydrocyclone with high turbulence levels [14]. It is noteworthy that the centrifugal classifier can be viewed as a device with dynamic components in the cyclone separator, and the flow characteristics are similar. Johansson and Evertsson studied the centrifugal air classifier in the aggregate industry by adopting RSM in their numerical simulation [15].

In order to evaluate the performance of a centrifugal classifier, it is necessary to obtain detailed information about the flow field. Some experimental studies on the flow characteristics inside the classifier have been carried out with LDV (Laser Doppler Velocimetry) and PIV (Particle Image Velocimetry). LDV measurements were conducted to study the effect of structural variations of the rotor cage on flow field characteristics inside the centrifugal classifier [16,17]. The velocity field was measured using PIV, and a lab-scale air classifier was established to study the classification mechanism of the commercial classifier [18]. The results show that useful information such as vortex location, separation performance, and flow field could be explained by the flow characteristics of the classifier parts. Moreover, interactions between gas and particles were studied in detail through the two-phase velocity measurements. Homann et al. looked at the effect of high Reynolds number turbulence on the force exerted on a single particle. The interaction between the fluid flow and the particles was investigated [19]; the drag force on the particle according to the turbulence intensity was measured, and was satisfactorily explained by a drag model similar to that of Schiller and Naumann [20].

In this work, numerical simulation was performed to investigate the effect of the design parameters on particle separation performance. Specifically the ring-shaped baffle was designed to block a specific area of air flow between guide vanes and the rotor cage. The effects of the baffle on the flow and particle separation in the centrifugal classifier were investigated. The RSM and a multiple reference frame (MRF) were employed to describe air flow, and DPM was adopted to predict particle motion in the classifier. The numerical results were validated by experiments using an industrial centrifugal classifier. Finally, the design parameters necessary for separating particles with a cut size of 4.38 µm were investigated.

## 2. Centrifugal Classifier

**Figure 1:**
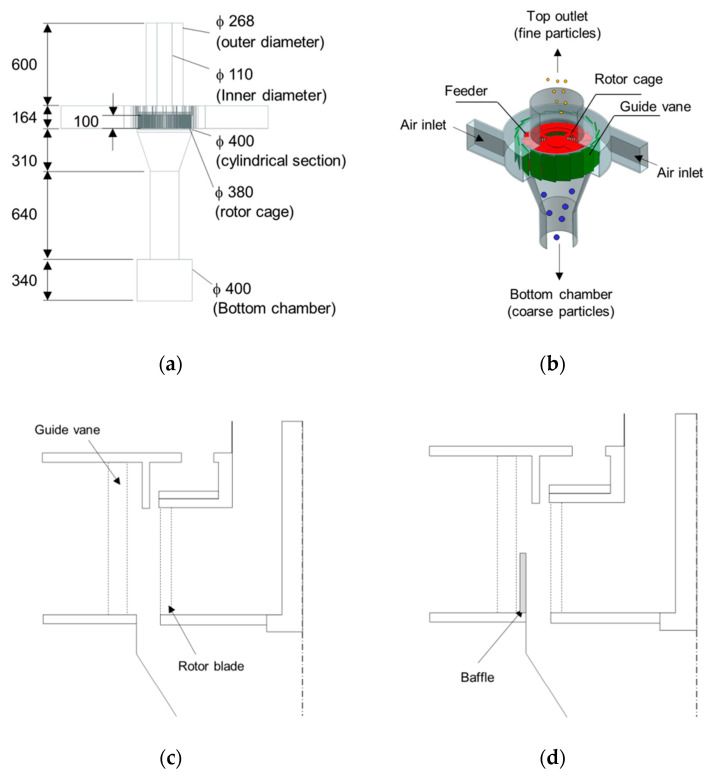
Schematic of the centrifugal classifier: (**a**) Dimensions; (**b**) Geometry; (**c**) Type 1 (baseline); (**d**) Type 2 (with the baffle).

A centrifugal classifier is an apparatus that separates a mixture of fine and coarse particles using the balance of three forces: drag, centrifugal, and gravity. Under the influence of the three forces, the fine and coarse particles are separated into different positions. The centrifugal classifier consists of the feed, inlet, guide vane, rotor cage, top outlet, and bottom chamber. Schematics of the centrifugal classifier are displayed in Figure 1. The mixture of coarse and fine particles is separated according to the following steps:

(1) The blower, which is connected at the top outlet, discharges air to the outside at a specific mass flow rate, and generates a negative pressure inside the classifier. Therefore, air is introduced through the inlet. Due to the cylindrical geometry and tangential velocity field, a high swirling flow is induced in the classifier.
(2) The mixture particles are supplied from the feeder and fall onto the rotor cage. A rotor cage with 90 blades is rotated by the motor. The rotor cage is equipped with ribs for better distribution of particles. The particles are thrown towards the classifier wall by the rotation of the rotor cage.
(3) The coarse particles are blocked by the blades and are not allowed to flow into the inner region. Therefore, most of them fall into the bottom chamber. As the rotational speed of the rotor cage increases, coarse particles collide more often with the blades.
(4) Meanwhile, due to the low inertia, the fine particles move with the air flow and pass through the blades of the rotor cage and exit through the top outlet.
(5) In this work, the effect of a ring-shaped baffle on the particle separation was investigated. As shown in Figure 1d, the baffle partially blocks this airflow from the guide vanes to the rotor cage. The baffle generates a strong velocity field locally in the direction of the rotor cage, allowing unseparated particles to flow back into the rotor cage.

## 3. Numerical Simulation

A three-dimensional numerical simulation was conducted to investigate the flow field and particle motion in the centrifugal classifier. RSM and MRF were used to describe the air flow, and DPM was adopted for the particle behavior. The numerical calculations were solved with the commercial CFD solver (ANSYS Fluent 19.2, Canonsburg, PA, USA).

### 3.1. Air Flow Field

In the centrifugal classifier, a complex three-dimensional turbulent flow, with high swirl, and very large curvature of streamline and rotation, is generated. In essence, all turbulence is anisotropic, but turbulent viscosity is assumed to be isotropic in the eddy viscosity models, such as k–ε and k–ω. In the literature [21,22], numerical simulations were performed on a stationary cyclone separator without dynamic rotating parts; however, the flow inside the centrifugal classifier also has similar features of swirling flow to separate particles. It has been proven that RSM is the most suitable turbulence model because it can provide much better accuracy for high swirling flow [4,12,15,23,24]. 

Reynolds averaged Navier–Stokes (RANS) equations in three dimensions can be expressed as:

(1) $$\frac{\partial \rho }{\partial t}+\frac{\partial \left(\rho u_{i}\right)}{\partial x_{i}}=0$$

(2) $$\frac{\partial \left(\rho u_{i}\right)}{\partial t}+\frac{\partial \left(\rho u_{i}u_{j}\right)}{\partial x_{j}}=-\frac{\partial p}{\partial x_{i}}+\frac{\partial }{\partial x_{j}}\left[\mu \left(\frac{\partial u_{i}}{\partial x_{j}}+\frac{\partial u_{j}}{\partial x_{i}}\right)-\frac{2}{3}\frac{\partial u_{k}}{\partial x_{k}}\delta_{ij}\right]+\frac{\partial }{\partial x_{j}}\left(\tau_{ij}\right)+\rho g$$

where the Reynolds stress tensor can be defined as:

(3) $$\tau_{ij}=-\rho \bar{u_{i}^{\prime }u_{j}^{\prime }}$$

In this work, the RSM was adopted as a closure model to solve the Reynolds stress tensor, which is modeled using the eddy viscosity ($\mu_{T}$) according to the Boussinesq hypothesis:

(4) $$-\rho \bar{u_{i}^{\prime }u_{j}^{\prime }}=\mu_{T}\left(\frac{\partial u_{i}}{\partial x_{j}}+\frac{\partial u_{j}}{\partial x_{i}}-\frac{2}{3}\frac{\partial u_{k}}{\partial x_{k}}\delta_{ij}\right)-\frac{2}{3}\rho k\delta_{ij}$$

The transport equation for the Reynolds stress is written as:

(5) $$\frac{\partial \left(\rho \bar{u_{i}^{\prime }u_{j}^{\prime }}\right)}{\partial t}+\frac{\partial \left(\rho u_{k}\bar{u_{i}^{\prime }u_{j}^{\prime }}\right)}{\partial x_{k}}=D_{ij}+P_{ij}+\Pi_{ij}+\varepsilon_{ij}$$

where the diffusion term ($D_{ij}$), the stress production term ($P_{ij}$), the pressure-strain term ($\Pi_{ij}$), and the dissipation term ($\varepsilon_{ij}$) are as follows:

$$D_{ij}=-\frac{\partial }{\partial x_{k}}\left[\rho \bar{u_{i}^{\prime }u_{j}^{\prime }u_{k}^{\prime }}+\left(\bar{p^{\prime }u_{j}^{\prime }}\right)\delta_{ik}+\left(\bar{p^{\prime }u_{i}^{\prime }}\right)\delta_{jk}-\mu \left(\frac{\partial u_{i}^{\prime }u_{j}^{\prime }}{\partial x_{k}}\right)\right]\;P_{ij}=-\rho \left(\bar{u_{i}^{\prime }u_{k}^{\prime }}\frac{\partial u_{j}}{\partial x_{k}}-\bar{u_{j}^{\prime }u_{k}^{\prime }}\frac{\partial u_{i}}{\partial x_{k}}\right)\;\Pi_{ij}=P\bar{\left(\frac{\partial u_{i}^{\prime }}{\partial x_{j}}\frac{\partial u_{j}^{\prime }}{\partial x_{i}}\right)}\;\varepsilon_{ij}=-2\mu \bar{\frac{\partial u_{i}^{\prime }}{\partial x_{j}}\frac{\partial u_{j}^{\prime }}{\partial x_{k}}}$$

### 3.2. Particle Motion

Particle motion in the fluid flow in the centrifugal classifier was predicted using the Eulerian–Lagrangian approach, which treats the air flow as a continuous phase, and the particles as a dispersed phase. The Li_2_CO_3_ particles were treated as the discrete phase traveling in the continuous fluid phase. The particles were sufficiently diluted that they did not affect the air flow, and particle-to-particle interactions were not considered. The forces acting on the particles resulted from the difference in velocity between the particles and the fluid. The equation of motion of the particles is expressed as:

(6) $$\frac{du_{p}}{dt}=F_{D}\left(u-u_{p}\right)+\frac{g\left(\rho_{p}-\rho \right)}{\rho_{p}}$$

where $u_{p}$ is the particle velocity, u is the fluid velocity, $\rho_{p}$ is the density of the particles, ρ is the density of the fluid, and $F_{D}$ is the drag force exerted on the particles.

The drag force per unit mass is calculated as:

(7) $$F_{D}=\frac{18\mu }{\rho_{p}d_{p}^{2}}\frac{C_{D}Re_{p}}{24}$$

The coefficient of drag force ($C_{D}$) is [10]:

(8) $$C_{D}=\left\{ \begin{array}{ll} \frac{24}{{Re}_{p}} & {Re}_{p}\leq 1\\ \frac{24}{{Re}_{p}}\left(1+0.15{Re}_{p}{}^{0.687}\right) & 1\leq {Re}_{p}\leq 1000\\ 0.44 & 1000\leq {Re}_{p} \end{array}\right.$$

where *Re_p_* is the Reynolds number of the particles. The second formula was chosen because the range of Reynolds number of the particles was 1 < *Re_p_* < 500. The Reynolds number of the particles is given by:

(9) $$Re_{p}=\frac{\rho \left|u-u_{p}\right|d_{p}}{\mu }$$

Actual turbulence flow applies random movement to particles, but the RANS model cannot solve all small turbulence eddies. Particle trajectories are affected by turbulent velocity fluctuations. The way to handle this is to use stochastic tracking [25]. The turbulence dispersion was used to accurately predict the particle separation with DPM [8,23,26].

### 3.3. Numerical Settings

In the basic test of the numerical analysis, two parameters were used. They were the rotor speed, which is the number of rotations of the rotor cage, and the mass flow rate at the top outlet, which is the fine particle outlet. In the preliminary test, steady-state analysis was successful at low mass flow rate and low rotor speed. However, as mass flow rate or rotor speed increased, unstable turbulence occurred, resulting in divergence or an oscillatory solution. Therefore, transient analysis was necessary because steady-state analysis cannot accurately capture the flow characteristics of the centrifugal classifier. The following solution strategy was used: in the first step, a steady-state solution using the k-epsilon model was used as an initial guess, to provide physically realistic conditions. In the second step, transient analysis was performed using RSM with the second order upwind discretization scheme. Up to five iterations were used to converge each time step, with the predefined residual criterion of 10^−3^. Numerical calculations were performed to obtain the steady-state solution. It is noteworthy that good initial conditions can reduce the number of time steps required to obtain the solution and enhance the solver stability. The time step Δ*t* was determined by taking the number of rotor blades into account. A total of 90 blades were installed in the rotor cage, and a single blade passage was divided into 20 steps. Therefore, the rotor cage rotates 360/90/20 = 0.2 degrees at each time step. For example, if the rotational speed of 1200 revs/min (= 7200 deg/s), the time step for 0.2 degrees of rotation is 0.2/7200 ~ 2.78 × 10^−5^ s; using this sufficiently small time step, it was possible to resolve the transient variables.

The PSD of the particles was described by the Rosin-Rammler function:

(10) $$1-Y=exp\left[-{\left(\frac{d}{\bar{d}}\right)}^{n}\right]$$

where *Y* is the volume fraction, smaller than a given diameter d, $\bar{d}$ is the mean diameter, and n is the spread parameter [27,28].

**Figure 2:**
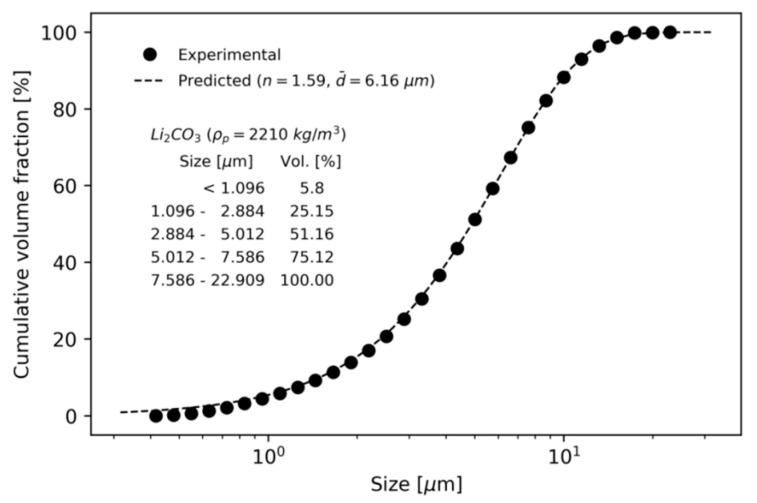
Particle size distribution of the mixed Li_2_CO_3_ particles.

The PSD of raw Li_2_CO_3_ particles was experimentally measured using a particle size analyzer (Mastersizer 2000, Malvern Instruments Ltd., Malvern, UK). The mean diameter and spread parameter were determined by curve fitting the experimental results to the Rosin-Rammler function. In Figure 2, the experimental and predicted cumulative volume fraction curves are compared. A minimum size of 0.407 µm, maximum size of 22.909 µm, mean size of 6.16 µm, and n value of 1.59 were used to define the PSD for the raw Li_2_CO_3_ particles.

The properties and boundary conditions used in numerical analysis are summarized in Table 1.

### 3.4. Numerical Domain

**Figure 3:**
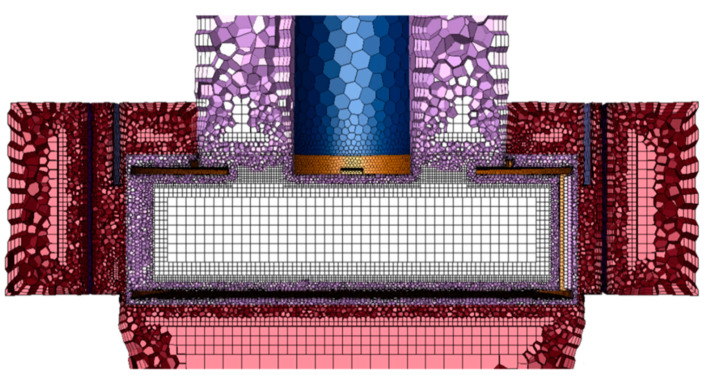
Mesh configuration used in the numerical simulation.

Figure 3 shows the mesh used in the numerical simulation; it consists of polyhedral and hexahedral cells, and three prism cells along the wall. Fine-enough cells were generated to resolve the flow variables, which change rapidly around the blade. In the mesh dependence test, the effects of the number of elements on the pressure drop and the particle separation performance were observed.

**Figure 4:**
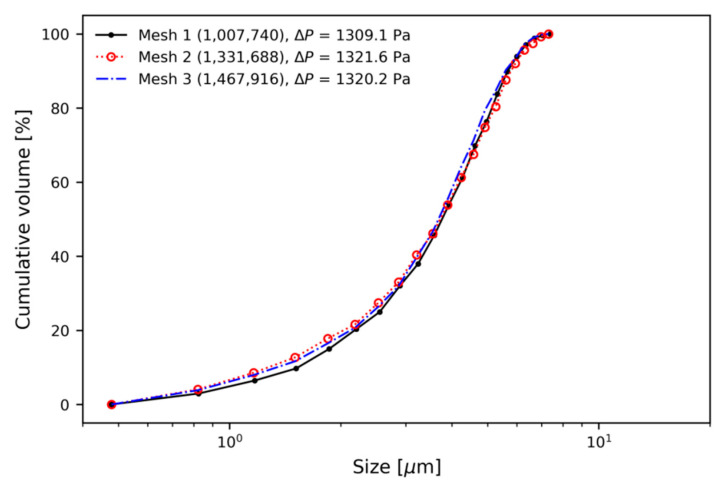
Mesh dependence test.

Using a fine mesh increases the computational resources needed to obtain the numerical solution. In particular, it is important to select the appropriate number of elements to investigate the combined effects of the various design parameters such as mass flow rate, rotor speed, and the ring-shaped baffle. From the numerical results, obtained by using different numbers of meshes, predictions of the pressure drop between inlet and outlet, and the particle separation performance, were found to be close to each other, without significant differences, as can be seen in Figure 4. There were small discrepancies in the separation performance for particles. Mesh 2 and Mesh 3 showed very similar results; finally, Mesh 2 (1,331,688 elements) was chosen to perform all numerical cases.

## 4. Particle Separation Performance

### 4.1. Pressure Field and Tangential Velocity Field

Numerical simulations on two types of classifier were performed: Type 1 without the ring-shaped baffle, and Type 2 with a 50 mm high baffle. A 50 mm high ring-shaped baffle can cover approximately half of the rotor cage area, because the inner height of the rotor blade is about 100 mm. This is shown in Figure 1a. The effects of the design parameters, such as mass flow rate and rotor speed, on the pressure fields and the tangential velocity fields were investigated. The mass flow rates were 0.2 and 0.4 kg/s, and the rotor speeds were 1200 and 1800 revs/min.

**Figure 5:**
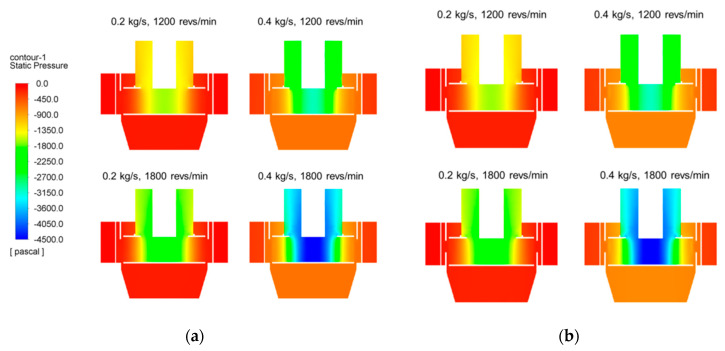
Pressure fields of two types of classifier: (**a**) Type 1; (**b**) Type 2.

Figure 5 shows the pressure fields in the vertical section of the classifier. There is no significant difference in pressure field between the two types of classifier. Negative pressure is generated at the center region by suction from the blower and rotation of the rotor cage. With increases in mass flow rate and rotor speed, there is an increase in the magnitude of the negative pressure. Higher pressure gradient is observed along the radial direction, and particles can move from the higher pressure region to the lower pressure region. This indicates that too strong a pressure gradient can be generated in the classifier, and particles can be discharged to the top outlet without sufficient separation.

**Figure 6:**
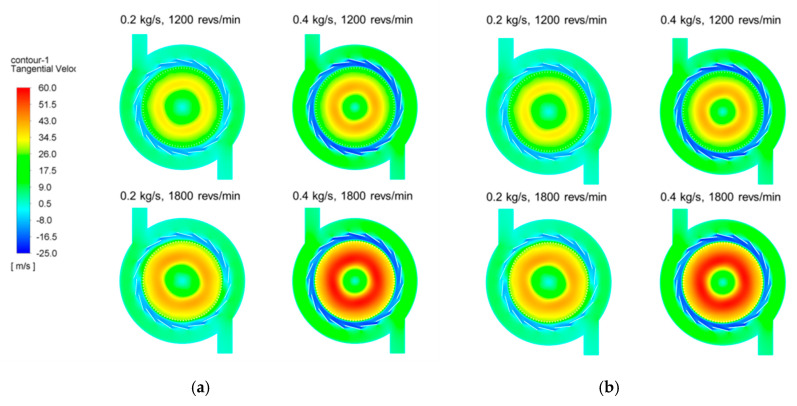
Tangential fields of two types of classifier: (**a**) Type 1; (**b**) Type 2.

Figure 6 shows the almost identical tangential velocity fields of the two classifiers at the horizontal section. Additionally, there is no noticeable distinction in tangential velocity field between the two types of classifiers. The magnitude of the tangential velocity increases with increasing mass flow and rotor speed, and high swirling flow is observed inside the rotor cage. The tangential velocity of the positive and negative values indicates that a non-uniform flow is generated inside the classifier. The tangential velocity varies remarkably along the radial direction, and has a positive value in the rotor cage region. However, the tangential velocity in the region between the guide vane and the rotor cage is negative. This indicates swirling flow in opposite directions; the feeder where the particles are injected is located in a region of very low tangential velocity, thus weakening the centrifugal force on the particles. Thus, coarse particles can move into the rotor cage, which means deterioration of the particle separation performance. It was possible to estimate the overall separation behavior of particles by observing the pressure field and tangential velocity field inside the classifier. The absence of significant changes in the pressure field and tangential velocity field in both types was justified in that the application of the baffle did not cause a major problem with the operating principle of the centrifugal classifier. The particle trajectories were observed for a more accurate prediction of separation performance.

### 4.2. Particle Separation

**Figure 7:**
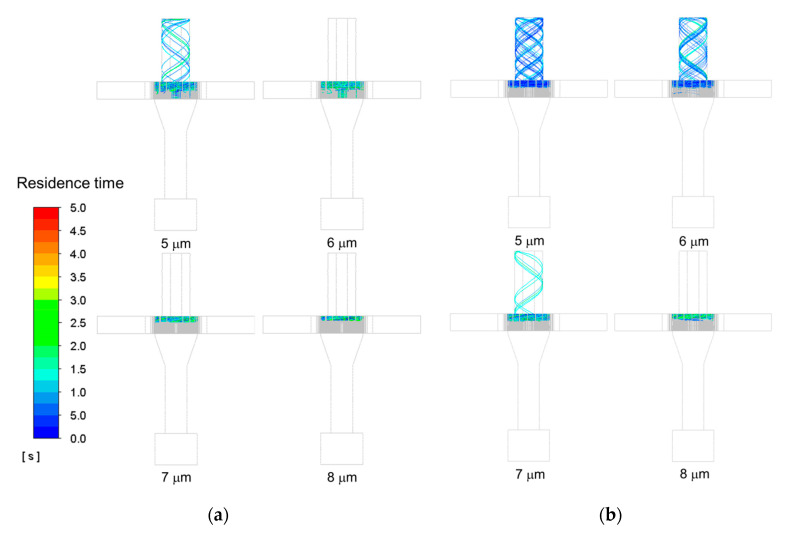
The particle trajectories of the different sizes at mass flow rate of 0.3 kg/s and rotor speed of 1800 revs/min: (**a**) Type 1; (**b**) Type 2.

**Figure 8:**
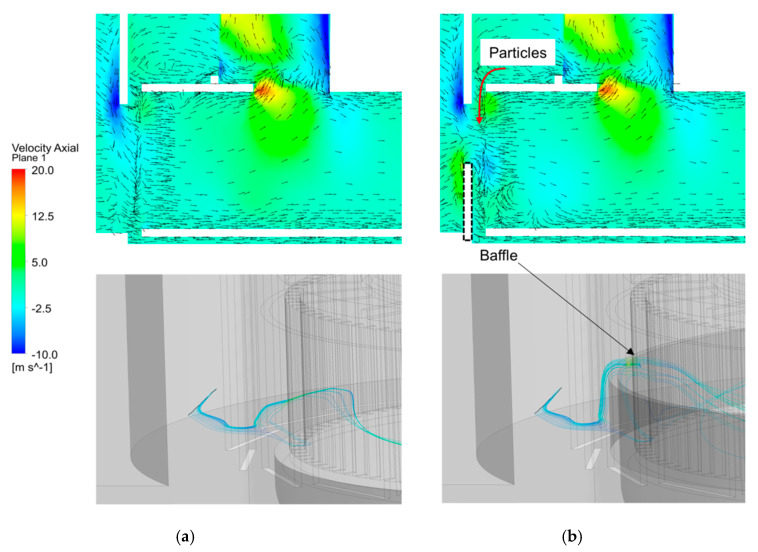
Velocity vectors and streamlines at mass flow rate of 0.3 kg/s and rotor speed of 1800 revs/min: (**a**) Type 1; (**b**) Type 2.

Figure 7 shows the calculated trajectories of the particles, with size ranging from 5 to 8 µm, in the Type 1 and Type 2. Mass flow rate of 0.3 kg/s and rotor speed of 1800 revs/min were used in the numerical analysis. It is shown that particles pass through the rotor cage region and escape through the top outlet in a spiral manner with the air flow. In Figure 7a, a particle size of 5 µm, is discharged through the top outlet in Type 1. In Figure 7b, however, particles ranging from 5 to 6 µm, and small amounts of 7 µm particles, exit through the top outlet. In both types of classifier, coarse particles larger than 7 µm do not enter the rotor cage region, and maintain a circular path for a long residence time. This can be explained by the drag force being greater than the centrifugal force. The role of the baffle is to change the movement direction of unseparated particles to the rotor cage region, increase the local air velocity in the separation zone, and increase particle separation throughput. Figure 8 shows the axial velocity vectors and streamlines near the rotor cage region. In Figure 8a, the air flow in the direction of the rotor cage, after passing through the guide vane, can be seen. Unseparated particles do not move to the rotor cage by the air flow, and fall in the direction of the bottom chamber by gravity. However, In Figure 8b, local upward air flows are generated by the installation of the baffle. This can be explained by the fact that more particles can be collected at the top outlet.

It is qualitatively illustrated that larger particles can be collected at the top outlet in Figure 7 and Figure 8. In addition, separation throughput and cut size (*d*_50_) were used to quantitatively evaluate how many particles were separated. The separation throughput is defined as the ratio of the number of particles separated through the top outlet, to the number of particles injected; the cut size is defined as the particle size for which the cumulative volume fraction has the value of 50%. 

**Figure 9:**
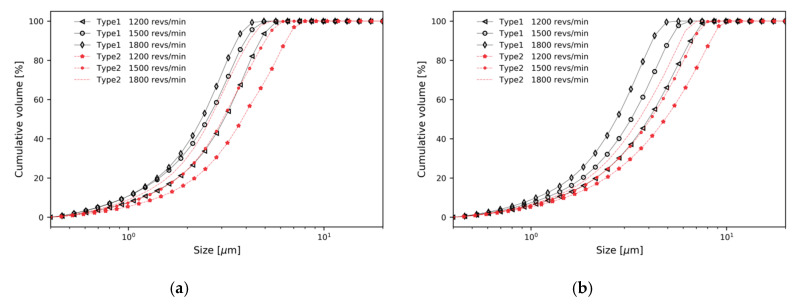
Cumulative volume distribution curves for two types of classifier; Type 1 and Type 2: (**a**) $\dot{m}$ = 0.2 kg/s; (**b**) $\dot{m}$ = 0.4 kg/s.

Figure 9 shows the PSD curves of cumulative volume for the two types of classifier and evaluates *d*_50_. For both types of classifier, particle separation performance was highly dependent on the design parameters such as mass flow rate and rotor speed. From Figure 9a to 9b, *d*_50_ increased as mass flow rate increased. This means that coarse and fine particles were discharged through the top outlet without sufficient separation by steep pressure gradient, as described in Figure 5. The steep pressure gradient means a strong drag force. As rotor speed increased, *d*_50_ decreased, which means that coarse particles were not discharged through the top outlet. This can be explained by two factors; the increase in tangential velocity and centrifugal force on the particles, as shown in Figure 6, and the blocking of coarse particles by the blades of the rotor cage. 

**Figure 10:**
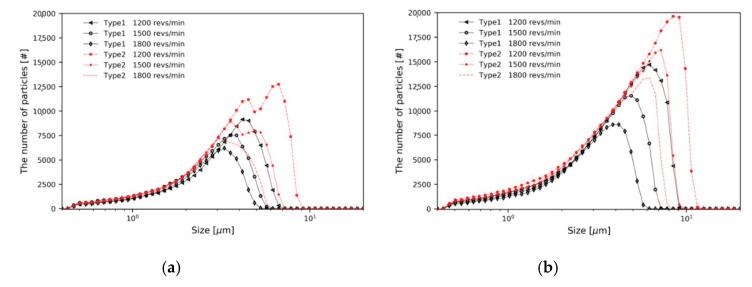
Frequency distribution of the number of particles for two types of classifier; Type 1 and Type 2: (**a**) $\dot{m}$ = 0.2 kg/s; (**b**) $\dot{m}$ = 0.4 kg/s.

To better compare the particle separation throughput, the frequency distribution of the number of particles is shown in Figure 10. In all cases, the number of particles separated through the top outlet is greater in Type 2 than in Type 1. Table 2 summarizes all numerical results for both classifiers, including cut size and separation throughput. In cases of Type 2 being compared to Type 1, it can be observed that a larger number of particles are separated through the top outlet. When the same number of particles, of the same distribution, is injected, an increase in throughput means an increase in the cut size. At the same mass flow rate and rotor speed, a significant contribution of the baffle is observed.

**Figure 11:**
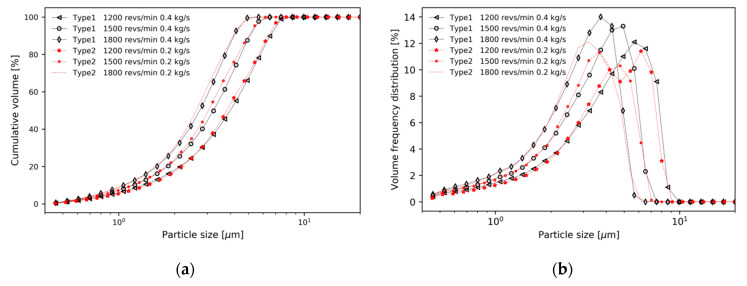
Comparison of Type 1 and Type 2: (**a**) Type 1; (**b**) Type 2.

Figure 11 compares the cumulative volume distribution and volume frequency distribution of Type 1 at 0.4 kg/s and Type 2 at 0.2 kg/s in Figure 9. Although the cut size of Type 2 is slightly smaller than Type 1, it can be seen that the two cases have similar values. This result by the baffle can be explained by the conservation of mass. In confined flows, assuming there is no additional accumulation of fluid within the volume, it follows that ${\dot{m}}_{i}={\dot{m}}_{o}$. Thus, conservation of mass requires

$$\rho_{i}A_{i}V_{i}=\rho_{o}A_{o}V_{o}$$

If the density of air remains constant, then $\rho =\rho_{i}=\rho_{o}$, and the equation above becomes

$$A_{i}V_{i}=A_{o}V_{o}$$

Therefore, the inlet velocity is *V_i_* = *A_o_V_o_/A_i_* [29]. If there is no difference in the mass flow rate at the top outlet, and the air inflow area has been reduced by the baffle, the velocity is inversely proportional by this equation, the air velocity of Type 2 locally increases compared to the air velocity of Type 1. This leads to an increase in local drag force acting on the particles, and eventually *d*_50_ increases. As shown in Figure 11, if the mass flow rate of the top outlet of Type 1 is twice the mass flow rate of that of Type 2, the inlet area of Type 2 is half of that of Type 1, so the velocity of air flowing from the inlet to the guide vane is the same: 

Type1:

$$2\rho {\left(A_{i}\right)}_{Type1}{\left(V_{i}\right)}_{Type1}=0.4\;{\left(V_{i}\right)}_{Type1}=0.2/\rho {\left(A_{i}\right)}_{Type1}$$

Type2:

$$2\rho {\left(A_{i}\right)}_{Type2}{\left(V_{i}\right)}_{Type2}=2\rho ({\left(A_{i}\right)}_{Type1}/2){\left(V_{i}\right)}_{Type2}=0.2\;{\left(V_{i}\right)}_{Type2}=0.2/\rho {\left(A_{i}\right)}_{Type1}$$

Assuming other conditions are the same, the same air velocity means the same drag force by Equations (7)–(9). This is a theoretical explanation to why *d*_50_ has similar values. Of course, the baffle changes the direction of air flow, so there may be a slight difference between the two types.

As explained earlier, the blower connected to the top outlet discharges air, which leads to air inflow at the inlet side. The baffle covers some area of the guide vanes, thus changing the direction and velocity of air entering through the guide vanes. This change affects the number of particles, which is separated through the top outlet and *d*_50_. The cut size and number of particles can be changed by adjusting only the rotor speed and mass flow rate, but the baffle has the effect of expanding the function of the blower. At the same mass flow rate and rotor speed, Type 2 increased the velocity in the separation zone, causing the increase of air drag force.

### 4.3. Experimental Settings

**Figure 12:**
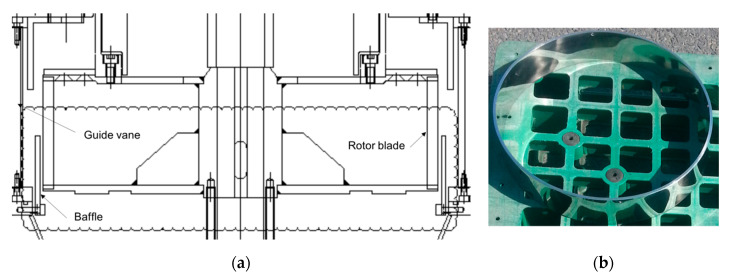
Centrifugal classifier with baffle: (**a**) Schematic of the centrifugal classifier with baffle; (**b**) Baffle.

Experiments were performed using a commercial centrifugal classifier (N-20, Seishin Co.), which has straight rotor blades [11] and traditional guide vanes without axial inclined angle [8]. The performance of the rotor and blower used in the experiment is summarized in Table 3. The ring-shaped baffle was manufactured with a height of 48 mm, an outer diameter of 396 mm, and an inner diameter of 386 mm. Figure 12 shows the design drawing of the ring-shaped baffle and the fabricated baffle. The baffle was screwed between the guide vane and the rotor inside the centrifugal classifier. Li_2_CO_3_ particles with PSD in Figure 2 were used as raw material particles.

First, to compare the results under the same conditions for Type 1 and Type 2, the experiment was carried out at a rotor speed of 1800 revs/min and a mass flow rate of 0.25 kg/s. Second, was to compare the condition where the mass flow rate of Type 2 was about half the mass flow rate of Type 1. Type 1 had a rotor speed of 3200 revs/min and a mass flow of 0.55 kg/s, and Type 2 had a rotor speed of 3000 revs/min and a mass flow of 0.25 kg/s. This was a range not included in the numerical analysis.

### 4.4. Experimental Results

The volume fraction PSD of the separated particles was determined using a Mastersizer 2000 laser diffraction particle size analyzer, with isopropyl alcohol as a dispersant. The number of particles obtained from the numerical results was converted to the volume fraction to compare with the experimental results.

**Figure 13:**
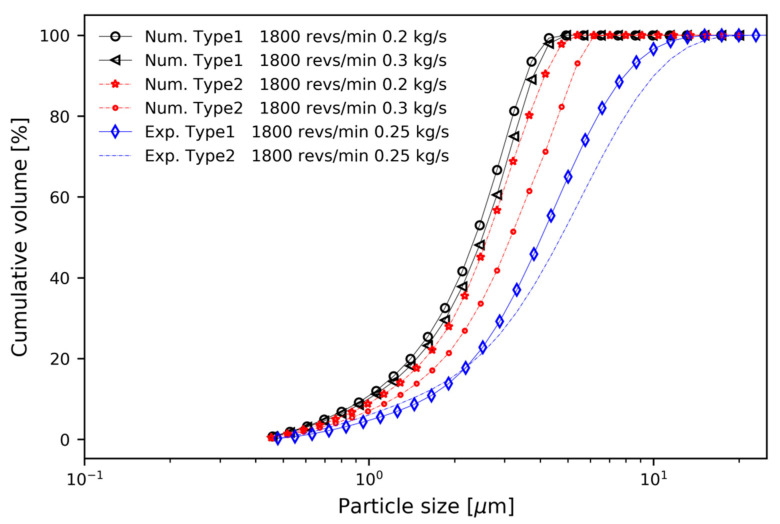
Comparison of experimental and numerical results.

Figure 13 shows a comparison of the experimental and numerical results of particle separations at the mass flow of 0.2 kg/s(Num.), 0.25 kg/s (Exp.), and 0.3 kg/s(Num.), and rotor speed of 1800 revs/min. The cumulative volume distribution curve is shown in Figure 13, but the slope of the experimental data at a mass flow rate of 0.25 kg/s is much smaller than that of the numerical analysis at mass flow rates of 0.2 kg/s and 0.3 kg/s. Although the values of the numerical prediction are different from the experimental data, the pattern is the same in Figure 13. In the numerical analysis, when the rotor speed was the same, the slope of Type 1 was steeper than that of Type 2. Even in the experiment, Type 1 had a steeper slope than Type 2. 

**Figure 14:**
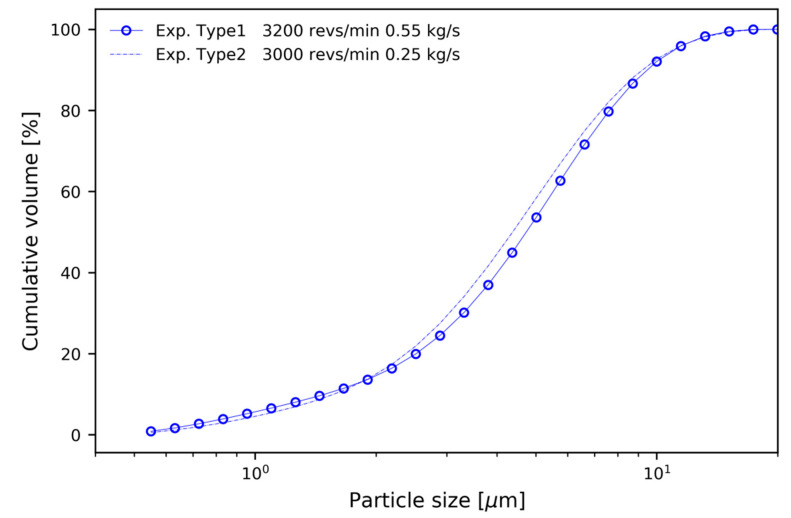
Comparison of experimental results of Type 1 and Type 2.

Figure 14 shows the comparison of cumulative volume distribution curves for Type 1 and Type 2 experiments, without numerical analysis. The experimental conditions for Type 1 were a mass flow of 0.55 kg/s and a rotor speed of 3200 revs/min, and for Type 2 a mass flow of 0.25 kg/s and a rotor speed of 3000 revs/min. The rotor speed of Type 1 and Type 2 was similar, and the mass flow rate of Type 2 was about half of that of Type 1. As expected from the numerical analysis, the two had similar values. Table 4 summarizes the results of the comparative experiment.

**Figure 15:**
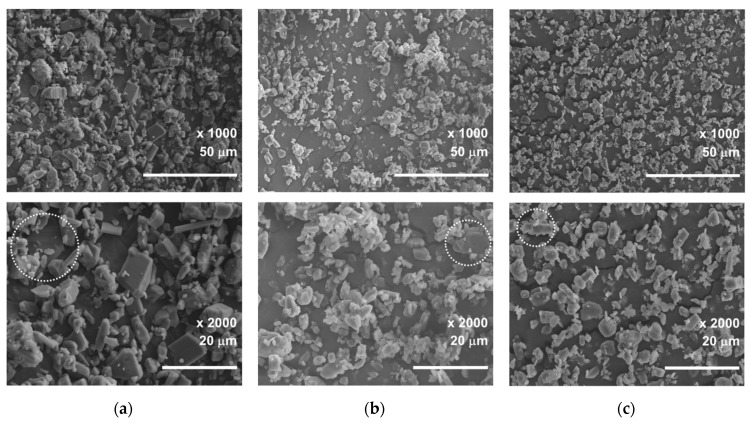
Microphotograph of the three samples visualized by FE-SEM: (**a**) Raw particles; (**b**) Type 1; (**c**) Type 2.

For the visual evaluation, the size and morphology of the particles were analyzed using a field emission scanning electron microscope (FE-SEM) as shown in Figure 15. Three samples were prepared randomly: raw Li_2_CO_3_ particles and separated particles, without baffle and with baffle. The point is, that by combining the parameters the desired grain cut size is obtained. The maximum size of 17 µm, can be observed in Figure 15a. The maximum sizes are about 10 µm in Figure 15b and 8 µm in Figure 15c.

The cut size can be larger in experiments than numerical analysis, because there are many factors that affect particle separation as well as rotor speed, mass flow rate, and baffle. These may include, experiment temperature, humidity, aging parts, fluctuations in raw materials, raw material input, fluctuations between workers, and so on. In addition, the conditions within the numerical analysis and the actual experimental environment are different, and the reality that can be expressed through numerical analysis is limited. A significant part of the prediction discrepancies can be attributed to the lesser degree of one-way coupling DPM; which does not account for the particle–fluid and particle–particle interactions. Moreover, as the particle size increases, an assumption of spherical particles may lead to deviations between numerical and experimental results, as shown in Figure 15. The one-way coupling has an advantage in calculation time, but if the particle size is large enough to affect the flow, the calculation accuracy may be relatively lower than two-way or four-way techniques. In the coupling study of particle and gas flow in a cyclone [30], when the swirl flow was affected by the particles, and the turbulence was damped, the separation performance was decreased. Furthermore, numerical predictions do not account for the important characteristics of Li_2_CO_3_ particles, such as agglomeration between particles and absorption of environmental moisture. Since the laser diffraction method is based on a simple equivalent sphere concept, it is not fully sufficient to describe the irregularly shaped particles, as shown in Figure 15.

## 5. Conclusions

In this work, numerical simulations of the flow field and particle motion of a centrifugal classifier were performed. In spite of the relatively simple geometries of the centrifugal classifier, the flow pattern and particle separation were complex. The swirling flow was captured by RSM, and particle motion and PSD were well-predicted by the DPM. 

The effects of the design parameters, such as mass flow rate and rotor speed, on the separation performance were investigated by numerical simulation. In addition, a ring-shaped baffle was designed to increase particle separation throughput by changing the direction of particle flow and increasing the local air velocity acting on the particles. Increasing the air velocity increases drag force acting on the particles, which has the effect of partially expanding the blower’s function. 

Then, experiments using a commercial centrifugal classifier were conducted to validate the numerical results. FE-SEM images of the separated particles proved the effects of the designed parameters. The objective of producing high quality lithium-ion batteries was successfully achieved by using the centrifugal classifier. In the separation experiment of lithium carbonate, conducted in this paper, a cut size of 4.04 µm was achieved with a combination of rotor speed and mass flow rate, and a cut size of 4.38 µm with a combination including the baffle. 

## Figures and Tables

**Table 1:** Properties and boundary conditions used in the numerical studies.

**Properties**	Air	Density: ρ = 1.225 kg/m^3^ Viscosity: μ = 1.7894 × 10^−5^ kg/m/s
	Li_2_CO_3_ Particles	Density: ρ_p_ = 2110 kg/m^3^ Size Range: 0.407 to 22.909 μm Mass Flow Rate: 0.013 kg/s
**Boundary Conditions**	Inlet	Pressure: 0 Pa Turbulence intensity: 3% Hydraulic diameter: 0.09812 m
	Top outlet	Mass flow outlet: 0.2, 0.3, 0.4 kg/s
	Rotor cage	Rotor speed: 900, 1200, 1500, 1800 revs/min

**Table 2:** Summary of numerical results.

No.	Mass Flow Rate [kg/s]	Rotor Speed [revs/min]	Type 1		Type 2	
			*d*_50_ [µm]	Throughput [–]	*d*_50_ [µm]	Throughput [–]
1	0.2	900	4.3	99,122	5.7	117,096
2	0.2	1200	3.4	92,416	4.2	111,694
3	0.2	1500	2.8	93,085	3.3	102,189
4	0.2	1800	2.6	73,868	2.9	91,400
5	0.3	900	4.6	100,034	6.0	131,068
6	0.3	1200	3.9	110,097	5.0	121,348
7	0.3	1500	3.3	96,866	4.2	118,323
8	0.3	1800	2.8	78,432	3.5	109,714
9	0.4	900	5.4	131,287	6.4	136,131
10	0.4	1200	4.4	120,295	5.3	130,655
11	0.4	1500	3.6	115,356	4.5	124,680
12	0.4	1800	3.0	101,777	3.9	121,627

**Table 3:** Rotor(N-20) and Blower.

Classifier N-20(Rotor)		Blower		
INV[Hz]	Motor [rpm]	INV[Hz]	Motor [rpm]	Mass Flow Rate [kg/s]
10	583	10	295	0.10
15	875	15	442	0.15
20	1167	20	590	0.21
25	1458	25	737	0.26
30	1750	30	884	0.31
35	2042	35	1032	0.36
40	2333	40	1179	0.41
45	2525	45	1327	0.46
50	2917	50	1474	0.51
55	3208	55	1622	0.56
60	3500	60	1769	0.62

**Table 4:** Summary of the comparative experiment.

			Type 1	Type 2
No.	Mass Flow Rate [kg/s]	Rotor Speed [revs/min]	*d*_50_ [µm]	*d*_50_ [µm]
1	0.25	1800	4.04	
	0.25	1800		4.73
2	0.55	3200	4.74	
	0.25	3000		4.38

## Nomenclature

***p***	**Fluid Pressure [Pa]**
*g*	Gravitational acceleration [m/s^2^]
*F_D_*	Drag force [N/m^3^]
C_D_	Drag coefficient of fluid [–]
*Re_p_*	Reynolds number of the particle
*u*	Fluid mean velocity [m/s]
*u’*	Fluctuant fluid velocity [m/s]
*u_i_*	Fluid velocity to i-direction (i = x, y, z) [m/s]
*u_j_*	Fluid velocity to j-direction (i = x, y, z) [m/s]
*u_p_*	Particle velocity [m/s]
*t*	Time [s]
Greek Letters	
*δ_ij_*	Kronecker delta [–]
*μ*	Fluid dynamic viscosity [kg-m/s]
*ρ_a_*	Fluid density [kg/m^3^]
*ρ_p_*	Particle density [kg/m^3^]

## Abbreviations

The following abbreviations are used in this manuscript:

PSD	Particle size distribution
CFD	Computational fluid dynamics
RSM	Reynolds stress model
LES	Large eddy simulation
DPM	Discrete phase model
LDV	Laser doppler velocimetry
PIV	Particle image velocimetry
MRF	Multiple reference frame
RANS	Reynolds averaged Navier–Stokes
FE-SEM	Field emission scanning electron microscope
